# Opportunistic DF-AF Selection Relaying in Hybrid Wireless and Power Line Communication for Indoor IoT Networks

**DOI:** 10.3390/s21165469

**Published:** 2021-08-13

**Authors:** Hoang Thien Van, Quyet-Nguyen Van, Danh Hong Le, Hoang-Phuong Van, Jakub Jalowiczor, Hoang-Sy Nguyen, Miroslav Voznak

**Affiliations:** 1 The Saigon International University (SIU), Ho Chi Minh City 700000, Vietnam; vanthienhoang@siu.edu.vn; 2 Faculty of Technology, Dong Nai Technology University, Dong Nai 760000, Vietnam; nguyenvanquyet@dntu.edu.vn; 3 Van Hien University, Ho Chi Minh City 700000, Vietnam; danhlh@vhu.edu.vn; 4 Faculty of Engineering and Technology, Thu Dau Mot University, Binh Duong 750000, Vietnam; 5 Faculty of Electrical Engineering and Computer Science, VSB—Technical University of Ostrava, 17, Listopadu 2172/15, 708 00 Ostrava, Czech Republic; jakub.jalowiczor@vsb.cz (J.J.); miroslav.voznak@vsb.cz (M.V.); 6 Faculty of Information Technology, Robotics and Artificial Intelligence, Binh Duong University, Binh Duong 750000, Vietnam; nhsy@bdu.edu.vn

**Keywords:** hybrid wireless and power line communication, wireless power transfer, energy harvesting, log-normal fading, opportunistic decode-and-forward and amplify-and-forward selection relaying, successful transmission probability

## Abstract

This manuscript investigates the system performance of hybrid wireless and power line communication networks for indoor Internet of Things applications. Differentiating itself from the existing literature, the performance of the direct link and dual-hop energy harvesting relay-aided links is analyzed under the condition of indoor fading modeled by log-normal distribution. Moreover, the manuscript presents the analytical expressions of the successful transmission probability of the deployed opportunistic decode-and-forward and amplify-and-forward relay selection scheme, and validates them with Monte Carlo simulations. Moreover, the impact of different system parameters on the successful transmission probability is revealed. For the considered hybrid system, in general, the opportunistic decode-and-forward relaying scheme outperforms the opportunistic amplify-and-forward relaying scheme. As importantly, increasing the source to relay distance and power splitting ratio over certain limits significantly deteriorates the system performance, indicated by the decrease in the successful transmission probability.

## 1. Introduction

The fifth-generation (5G) wireless communication is founded on the 4G/IMT-Advanced standards to serve the forecasted tens or even up to hundreds of billions of connected devices due to the continuous growth of state-of-the-art personal communication applications [1,2]. In the upcoming Internet of Things (IoT) era, it is predicted in [3,4,5] that by the year of 2030, there would be approximately 80 billion connected devices in a network and an individual can connect up to 20.5 billion devices simultaneously. Therefore, the expectation for the 5G communication has been that it can significantly improve the bandwidth, the data transmission rates, and the connectivity reliability and extend the network coverage while offering remarkable reductions in energy consumption and signal latency [3].

To enable long-lasting communication networks, several research studies have been conducted on self-sustaining simultaneous wireless information and power transfer (SWIPT) technology for energy harvesting (EH) from radio frequency (RF), firstly in [6] and ever since in [7,8,9]. There are two SWIPT protocols for the operation of receivers, namely time switching-based relaying (TSR) and power splitting-based relaying (PSR), which were proposed in [10,11]. In TSR mode, the receiver can switch between information decoding (ID) and EH. On the other hand, PSR mode enables the receiver to partition the signal power into two parts dedicated to ID and EH. In [12], the authors studied the trade-off between the transmission outage probability (OP) and the ergodic capacity, respectively, versus the amount of energy harvested at the receiver in TSR and PSR scenarios. The energy efficiency (EE) of the SWIPT was studied in [13,14] and the physical-layer security aspect was investigated in [15,16,17].

Furthermore, to improve the EE, the spectrum efficiency (SE), the data transmission rates, and the throughput and coverage range, the cooperative relaying network has been investigated in [18,19,20,21]. Indeed, considerable diversity can be gained by exploiting several intermediate relays to aid the data transmission from the source node to the destination node [22]. Hence, two main cooperative relaying protocols, namely amplify-and-forward (AF) and decode-and-forward (DF), were investigated in [23]. Furthermore, a so-called ODF-AF selection relaying protocol that enables relays to adaptively switch between DF and AF considering their local signal-to-noise ratio (SNR) was proposed and investigated in [24,25,26,27]. It was proven in [28,29] that the ODF-AF scheme outperforms the standalone ODF and OAF schemes in terms of the outage performance of the system.

From relays’ perspective, they can be operated in half-duplex (HD) and full-duplex (FD) modes. In the former, the relays are configured with one antenna utilizing the dedicated and orthogonal channels for re-transmitting data, while in the latter, with two antennas for data transmission within the same time slot and bandwidth. Furthermore, there is a hybrid HD-FD, which allows the opportunistic switch between the two aforementioned modes, and was proven to be able to deliver notably better performance, as studied in [30]. Wireless networks operating in FD mode can promisingly multiply the SE by two times and deliver significantly better network throughput in comparison with HD mode. Nevertheless, FD relays suffer self-interference because of the leaked signal between the two antennas [31], which inevitably degrades the system performance. Accordingly, in [32], the authors proposed different techniques to mitigate this loop interference for FD relaying networks along with their pros and cons.

As aforementioned, systems with higher diversity gain perform better thanks to the higher amount of independent fading signals that can be combined from multi-relay utilization [22,33], yet they face a higher risk of system degradation due to the higher level of inter-relay interference. To solve this, different relay selection (RS) schemes with their positive and negative effects on system performance were conducted in [34,35,36,37]. In particular, several different RS schemes for FD-AF cooperative networks were presented in detail in [34]. Paper [35] proposed a buffer-state-based RS scheme with the help of the Markov chain model. The authors in [36,37], respectively, studied the security improvement in DF cooperative relaying systems and the security–reliability trade-off of cognitive radio systems utilizing different RS schemes.

There are several studies about EH relaying SWIPT networks in the existing literature conducted over some prominent outdoor fading channel models, namely Nakagami-m, Rayleigh, and Rician. Nevertheless, for characterizing the shadowing effect of indoor scenarios owing to building walls, human body, and object mobilities, a so-called log-normal fading channel model was proven a better option [38,39,40,41]. The characteristics of common fading channel models along with their applications were investigated and compared in [42], and the appropriateness of the log-normal for indoor scenario modeling was proven in terms of small-scale fading and frequency of outage events. Additionally, there is paper [43], which studied the hybrid TSR-PSR protocol for EH networks, and paper [44], analyzing the performance of the two-hop AF relaying networks.

To efficiently promote the development of 5G networks for smart home and smart city applications, it is worth utilizing the existing power line communication (PLC) system that is present in every household. Indeed, PLC has regained the attention of the research community in recent years, being a well-founded medium for smart grids (SG) and IoT [45]. Besides the fact that PLC can notably reduce the installation cost, it can effectively establish communication with nodes that cannot be reached with RF due to the severe attenuation in the household setting. On the other hand, conventional PLC systems suffer from multipath fading effects and the in-line signal is degraded exponentially as the communication distance increases, which can be effectively tackled with the help of relay-aided networks [46]. Practically, because of the differences in the channel characteristics between the wireless and PLC networks, there is a need to implement dual-interface wireless–PLC relays as described in [47,48,49] for such hybrid wireless and PLC (HWP) networks to function. Research studies have proven that reliable signal transmission could be ensured even with a deterioration in quality on both links. Having deployed the dual-interface relays, it is then possible to apply all the aforementioned advantages of EH cooperative relaying networks on HWP, noting that, in the majority of studies, this has been achieved thanks to the help of time division multiple access (TDMA) schemes, as in [50,51,52,53].

With inspiration taken from the aforementioned studies, this manuscript focuses on the STP performance of the ODF-AF relaying selection in HD cooperative relaying networks given the indoor PLC condition making up the HWP, whose characteristics are modeled with log-normal fading channels. Subsequently, the main contributions of this manuscript are listed below:

- The STP for the direct link and ODF-AF relay-aided links of the dual-hop HD EH HWP over log-normal fading channels is analytically expressed.
- The STP and throughput performance of the ODF-AF selection relaying scheme in the HWP are analyzed and validated with Monte Carlo simulation results.

Moreover, it is worth noting the important notations utilized in this manuscript. Specifically, the probability density function (PDF) and the cumulative distribution function (CDF) of the log-normally distributed random variable (RV) *X* are, respectively, denoted as $F_{X}\left(z\right)=1-Q\left(\frac{10\ln{\left(10\right)}^{-1}\ln\left(z\right)-2\omega_{X}}{2\Omega_{X}}\right)$ and $f_{X}\left(z\right)=\frac{10\ln{\left(10\right)}^{-1}}{z\sqrt{8\pi \Omega_{X}^{2}}}\exp\left(-\frac{{\left(10\ln{\left(10\right)}^{-1}\ln\left(z\right)-2\omega_{X}\right)}^{2}}{8\Omega_{X}^{2}}\right)$ given the Gaussian Q-function being Q(·) with $Q\left(x\right)=\int_{x}^{\infty }\frac{1}{\sqrt{2\pi }}\exp\left(-\frac{t^{2}}{2}\right)dt$. Moreover, there is E, which represents the statistical mean operation.

Aside from the general Introduction in Section 1, Section 2 describes the system model with certain assumptions. In Section 3, the overall STP performance of the dual-hop HD EH HWP with ODF-AF relaying in EH-PSR protocol is derived. Accordingly, Section 4 reports the numerical results. Finally, Section 5 concludes the manuscript’s findings and suggests possible future works. Furthermore, the abbreviations used in this manuscript are listed above the References section for ease of lookup.

## 2. System Model

**Figure 1:**
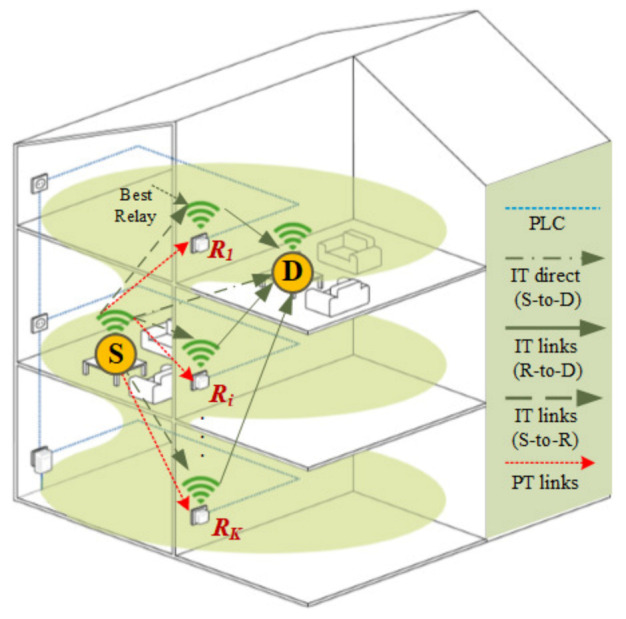
A typical HWP system with relaying nodes having both wireless and PLC interfaces. The relays scatter on different floors and rooms, and PLC relays are installed in electrical devices, which are connected with the power line.

In this study, a typical HWP system for indoor IoT is considered with dual-interface wireless–PLC relays integrated on all devices in the network, forming an Ad Hoc, as described in [47]. As mentioned earlier, PLC nodes will take the lead in establishing the communication among transceivers between several walls, and so do wireless nodes in case a lengthened power line notably downgrades the communication signal. As illustrated in Figure 1, a cooperative wireless relaying network is integrated into a PLC system for better communication between IoT devices, represented by a source (S), a destination (D), and a cluster (C) of *K* relays ($R_{i}$ with 1≤i≤K). It is worth noting that PLC, IT, and PT, respectively, stand for the power line communication network, information transmission, and power transmission.

In the proposed HWP system, the received signals at the relaying nodes of both the interfaces can be described as follows.

(1) $$\left[\begin{array}{c} y_{p}\left(t\right)\\ y_{w}\left(t\right) \end{array}\right]=\left[\begin{array}{cc} \sqrt{P} & 0\\ 0 & \sqrt{P} \end{array}\right]\left[\begin{array}{cc} h_{p} & 0\\ 0 & h_{w} \end{array}\right]\left[\begin{array}{c} x(t)\\ x(t) \end{array}\right]+\left[\begin{array}{c} n_{p}\left(t\right)\\ n_{w}\left(t\right) \end{array}\right],$$

where there are the transmitted symbol x(t), channel gains from transmitting to receiving nodes over PLC and wireless channels, $h_{p}$ and $h_{w}$, with noise, $n_{p}\left(t\right)$ and $n_{w}\left(t\right)$, and the transmission power *P*. In particular, for the HWP system, a module so-called signal decision processor (SDP) is deployed in the relaying node to evaluate and select the channel with the higher received SNR to forward the signal it received. Hence, the received SNR at the receiving node is expressed as follows.

(2) $$\gamma =\max\left\{Ph_{p}^{2}/n_{p}^{2},Ph_{w}^{2}/n_{w}^{2}\right\}.$$

For such a HWP system, it is obvious that there is a need to optimize both the PLC and the wireless channels. Within the scope of this study, it is assumed that the direct S–D link is under severe attenuation, and the communication is re-establishable neither by the PLC, due to lower received SNR, nor the direct wireless links, but solely via the cooperative relay-aided links. This assumption is equivalent to the wireless network in case there is either the coverage extension, where relays are utilized to establish the connection between significantly distant S–D [15,31], or when the direct S–D link is under a deep shadowing effect owing to the presence of the surrounding physical obstacles [54]. It is noteworthy that this setup has been utilized widely in the existing literature, with proven effectiveness in studying the cooperative networks and the potential diversity gain from such processes [15,31,54]. This setup is applicable even for cellular networks (LTE-Advanced), as in [55]. Furthermore, every terminal is aware of the channel state information (CSI) in advance and there is an ideal carrier and symbol synchronization. In addition, S is energized by a stable conventional power source $P_{S}$, and the *i*-th R is energized by the energy $P_{R_{i}}$ from the EH module. In addition, $n_{j}$ with $j\in \left\{r_{k},d\right\}$ is defined as the additive white Gaussian noise (AWGN) with zero mean and variance $N_{0}$ at the *i*-th relay and D, respectively.

It is assumed that the S–$R_{i}$ and $R_{i}$–D links are under the quasi-static block fading effect. This means that the channels remain constant over the block time, and they are independently and identically distributed (i.i.d.) following log-normal distribution from one block to another. As aforementioned, log-normal fading channels are utilized thanks to the appropriateness in modeling indoor scenarios with moving objects, furniture, and several walls. The S–$R_{i}$, $R_{i}$–D and S–D links are with channel coefficients *X*, *Y*, and *Z*, respectively, and are correspondingly distant $d_{X}$, $d_{Y}$, and $d_{Z}$ from each other. Similar to several studies dealing with log-normal fading channels [39], every communication node is equipped with one antenna, including the HD relays. Communication time is divided into slots given that, during a time slot interval, only a relay within C ($R_{i}\in C$) is selected for assisting the signal transmission from S.

Accordingly, the ${\left|X\right|}^{2}$, ${\left|Y\right|}^{2}$ and ${\left|Z\right|}^{2}$ are logically assumed as i.i.d. log-normal RVs, which are specified, respectively, with $LN\left(2\omega_{X},4\Omega_{X}^{2}\right)$, $LN\left(2\omega_{Y},4\Omega_{Y}^{2}\right)$, and $LN\left(2\omega_{Z},4\Omega_{Z}^{2}\right)$. It should be noted that both the $\omega_{j}$ and $\Omega_{j}^{2}$ are in decibels (dB), and they respectively represent the mean and the standard deviation of $10\log_{10}\left(j\right),j\in \left\{X;Y;Z\right\}$.

First of all, a direct transmission protocol is considered, in which all time slots of a signal block are dedicated to the signal transmission of the direct S–D link. Accordingly, D receives the signal described by the base band-equivalent discrete-time model as follows:

(3) $$y_{Z}=\sqrt{\frac{1}{d_{Z}^{m}}}Zs(t)+n_{d}\left(t\right),$$

where *s* indicates the narrow-band transmitted signal from S with zero mean, $E\left[{\left|s\right|}^{2}\right]=P_{S}$, and *m* is the path loss exponent.

Consequently, the SNR for the direct S–D link obtained by utilizing the zero-mean, circularly symmetric, complex Gaussian inputs is expressed as

(4) $$\gamma_{Z}=\Lambda \frac{{\left|Z\right|}^{2}}{d_{Z}^{m}},$$

where $\Lambda =\frac{P_{S}}{N_{0}}$.

In addition, the instantaneous capacity of the S–D link is

(5) $$C_{s,d}=W\log_{2}\left(1+\gamma_{Z}\right),$$

where *W* stands for the frequency bandwidth.

In the direct transmission protocol, thanks to the CDF of the log-normally distributed RV ${\left|Z\right|}^{2}$, the STP can be formulated as

(6) $$\begin{array}{cc} STP_{s,d}= & \mathrm{Pr}\left\{\gamma_{Z}\geq R_{Z}\right\}\\ = & 1-\mathrm{Pr}\left\{{\left|Z\right|}^{2}<\frac{R_{Z}}{\Lambda d_{Z}^{-m}}\right\}\\ = & Q\left(\frac{10\ln{\left(10\right)}^{-1}\ln\left(\frac{R_{Z}}{\Lambda d_{Z}^{-m}}\right)-2\omega_{Z}}{2\Omega_{Z}}\right) \end{array},$$

where $R_{Z}=2^{\frac{C_{th}}{W}}-1$.

Furthermore, the STP is respectively formulated for the AF and DF protocols as follows

(7) $$STP_{s,r,d}^{DF}=\mathrm{Pr}\left\{C_{s,r,d}^{DF}\geq C_{th}\right\},$$

and

(8) $$STP_{s,r,d}^{AF}=\mathrm{Pr}\left\{C_{s,r,d}^{AF}\geq C_{th}\right\},$$

where $C_{s,r,d}^{DF}=\min\left\{C_{r}\left(\gamma_{ri}^{DF}\right),C_{d}\left(\gamma_{di}^{DF}\right)\right\}$, and $C_{s,r,d}^{AF}=C_{d}\left(\gamma_{di}^{AF}\right)$. $C_{r}$ and $C_{d}$ are, respectively, the instantaneous capacities at the *i*-th R and D, with the corresponding SNR being $\gamma_{ri}$ and $\gamma_{di}$. $C_{th}$ is a to-be-specified threshold value.

In the context of cooperative relaying networks where every relay utilizes the ODF-AF selection relaying protocol, the decoding state of $R_{i}$, i∈1,⋯,K is denoted as $\chi_{i}$. If $\chi_{i}=0$, then $R_{i}$ utilizes the AF protocol for relaying the received signal. Otherwise, when $\chi_{i}=1$, the DF protocol is utilized. Accordingly, the condition for the best relay *k* to be selected is

(9) $$k=\arg\max_{i=1,2,\cdots ,N}\left\{\chi_{i}\min\left\{\gamma_{ri}^{DF},\gamma_{di}^{DF}\right\}+\left(1-\chi_{i}\right)\gamma_{di}^{AF}\;\right\}.$$

Accordingly, the STP of the ODF-AF selection relaying scheme can be obtained from

(10) $$\begin{array}{cc} STP_{k}^{ODF-AF}= & \left\{\chi_{k}\mathrm{Pr}\left\{\min\left\{C_{r}\left(\gamma_{rk}^{DF}\right),C_{d}\left(\gamma_{dk}^{DF}\right)\right\}\geq C_{th}\right\}\right\}+\left(1-\chi_{k}\right)\mathrm{Pr}\left\{C_{d}\left(\gamma_{dk}^{AF}\right)\geq C_{th}\right\}. \end{array}$$

**Remark** **1.** *STP is utilized to evaluate the system performance of the proposed relay-aided cooperative protocol. It is defined as the probability of a receiver succeeding in receiving packets from its corresponding transmitter within a time slot interval. Specifically, in this study, the transmission time is slotted, in which S, R, and D take turns to send their packets when each slot begins. Specifically, when S attempts to send some of its packets, if the instantaneous capacity is greater than a pre-specified threshold value, an acknowledgement signal (ACK) will be sent to S indicating that R has succeeded in receiving the packets. These packets are then removed from the queue at S. Otherwise, they remain on top of the queue. From R, they are eventually transmitted in a similar manner to D to accomplish one transmission circle. It is noteworthy that the STP formulation can be extended to multi-hop networks, given that every slot of the networks must be considered, instead of solely two slots, as in this dual-hop case.*

For the PSR protocol, the block time *T* is halved, with one half utilized for the signal transmission of the S–R link and the other for the R–D link. Within the first half interval, a portion of the signal power that R receives, being $\delta P_{S}$, is allocated for the EH module given the PS factor δ and 0≤δ≤1. The rest of the signal power, being $\left(1-\delta \right)P_{S}$, is utilized for signal transmission. In case δ=1, the system operates in full EH mode, and δ=0 in full ID mode.

Thus, within the first time slot, the EH module receives the input signal of

(11) $$\sqrt{\delta }y_{r}\left(t\right)=\sqrt{\delta }\sqrt{\frac{1}{d_{X}^{m}}}Xs\left(t\right)+n_{r}\left(t\right).$$

Moreover, the base-band signal at the information receiver, being $\sqrt{1-\delta }\gamma_{r}\left(t\right)$, is expressed for both AF and DF as

(12) $$\sqrt{1-\delta }y_{r}\left(t\right)=\sqrt{\left(1-\delta \right)}\sqrt{\frac{1}{d_{X}^{m}}}Xs\left(t\right)+n_{r}\left(t\right).$$

In this protocol, R only harvests, within each block interval, enough energy required to perform its relaying task [56] (Sec. III-B). Thus, when each time block ends, there is no energy remaining in R. This harvested energy during the first phase is obtained by

(13) $$E_{H}=\frac{\eta \delta P_{S}{\left|X\right|}^{2}T}{2d_{X}^{m}},$$

where 0≤η≤1 is the EH efficiency characterized by the property of the circuitry.

During the T/2 interval, all of the harvested energy is consumed by R to re-transmit the message from S to D with power $P_{R}$. Hence, the amount of harvested energy at the instantaneous T/2 time is

(14) $$E_{H}=P_{R}\frac{T}{2}.$$

As (13) is equal to (14), $\delta^{*}$ can be obtained as

(15) $$\delta^{*}=\frac{P_{R}}{\eta \delta P_{S}}\frac{d_{X}^{m}}{{\left|X\right|}^{2}}.$$

Accordingly, the relay’s transmitting power, as in [6,18], is given by

(16) $$P_{R}=\frac{\eta \delta P_{S}{\left|X\right|}^{2}}{d_{X}^{m}}.$$

**Remark** **2.** *It is noteworthy that the direct link and relay links are available for information transmission in the proposed relay-aided cooperative HWP system. One R with a sufficient harvested energy amount among ks is selected to establish the relay-aided link to substitute the deep faded direct link. Additionally, it is proven in [40] that the application of RS schemes in systems powered by an EH module can help to attain the maximum diversity gain amounting to the number of operated R nodes over the i.i.d. log-normal fading channel.*

## 3. Performance Analysis

In this section, the STP of the HD cooperative relaying HWP system over log-normal fading channels is investigated for the DF and AF protocols.

### 3.1. Opportunistic Decode-and-Forward (ODF) Relaying Scheme

Within the second time slot, as the name suggests, the signal from (16) is decoded, re-modulated, and then forwarded utilizing the harvested energy from (13). Thereby, in the HD-DF HWP system, at D, the received signal can be obtained from

(17) $$y_{d}\left(t\right)=\sqrt{\frac{1}{d_{Y}^{m}}}Y\bar{s}\left(t\right)+n_{d}\left(t\right),$$

where $\bar{s}$ is the narrow-band transmitted signal at *i*-th R with zero mean and $E\left[{\left|\bar{s}\right|}^{2}\right]=P_{R}$.

Combining (12), (16) and (17), one can express the SNRs at the *i*-th R and D, respectively, as

(18) $$\gamma_{ri}^{DF}=\frac{(1-\delta )\Lambda }{d_{X}^{m}}{\left|X\right|}^{2},$$

and

(19) $$\gamma_{di}^{DF}=\frac{\eta \delta \Lambda }{d_{X}^{m}d_{Y}^{m}}{\left|X\right|}^{2}{\left|Y\right|}^{2}.$$

In the HD-DF-PSR HWP system, the instantaneous capacity of the first and second links can be obtained from

(20) $$C_{j}=\frac{1}{2}W\log_{2}\left(1+\gamma_{ji}^{DF}\right),$$

where j∈{r,d}, and the HD relaying factor $\frac{1}{2}$.

**Theorem** **1.** *The STP of the HD-DF-PSR HWP system is formulated as*

(21) $$\begin{array}{cc} STP_{s,r,d}^{DF}= & Q\left(\frac{10\ln{\left(10\right)}^{-1}\ln\left(a_{1}\right)-2\omega_{X}}{2\Omega_{X}}\right)\\ \; & -\frac{10\ln{\left(10\right)}^{-1}}{\sqrt{8\pi \Omega_{X}^{2}}}\int_{a_{1}}^{\infty }\frac{1}{x}\exp\left(-\frac{{\left(10\ln{\left(10\right)}^{-1}\ln\left(x\right)-2\omega_{X}\right)}^{2}}{8\Omega_{X}^{2}}\right)\\ & \times \left(1-Q\left(\frac{10\ln{\left(10\right)}^{-1}\ln\left(a_{2}x^{-1}\right)-2\omega_{Y}}{2\Omega_{Y}}\right)\right)dx, \end{array}$$

*where $R_{th}=2^{2C_{th}/W}-1$, $a_{1}=d_{X}^{m}R_{th}/\left(1-\delta \right)\Lambda$, and $a_{2}=d_{X}^{m}d_{Y}^{m}R_{th}/\eta \delta \Lambda$.*

**Proof.**  With regard to (7), the STP is re-organized as

(22) $$\begin{array}{cc} STP_{s,r,d}^{DF}= & \mathrm{Pr}\left\{\min\left\{C_{r}\left(\gamma_{ri}^{DF}\right),C_{d}\left(\gamma_{di}^{DF}\right)\right\}\geq C_{th}\right\}\\ = & \mathrm{Pr}\left\{C_{r}\left(\gamma_{ri}^{DF}\right)\geq C_{th},C_{d}\left(\gamma_{di}^{DF}\right)\geq C_{th}\right\}\\ = & \underset{STP_{1}^{DF}}{\underset{︸}{\mathrm{Pr}\left\{C_{r}\left(\gamma_{ri}^{DF}\right)\geq C_{th}\right\}}}\cap \underset{STP_{2}^{DF}}{\underset{︸}{\mathrm{Pr}\left\{C_{r}\left(\gamma_{ri}^{DF}\right)\geq C_{th},C_{d}\left(\gamma_{di}^{DF}\right)<C_{th}\right\}}}. \end{array}$$

To calculate the STP in the HD-DF-PSR HWP system, two probability calculations are required. From (18) and (20), the first probability in (22) can be rewritten in detail as

(23) $$\begin{array}{cc} STP_{1}^{DF} & =\mathrm{Pr}\left\{\frac{1}{2}\log_{2}\left(1+\frac{\left(1-\delta \right)\Lambda {\left|X\right|}^{2}}{d_{X}^{m}}\right)\geq C_{th}\right\}\\ \; & =\mathrm{Pr}\left\{{\left|X\right|}^{2}\geq R_{th}\frac{d_{X}^{m}}{(1-\delta )\Lambda }\right\}\\ \; & =1-F_{X}\left\{a_{1}\right\}\\ \; & =Q\left(\frac{10\ln{\left(10\right)}^{-1}\ln\left(a_{1}\right)-2\omega_{X}}{2\Omega_{X}}\right) \end{array},$$

where there are the CDF of the log-normally distributed RV $X={\left|X\right|}^{2}$, $R_{th}=2^{2C_{th}}-1$, and $a_{1}=\frac{d_{X}^{m}R_{th}}{(1-\delta )\Lambda }$.

Likewise, (18)–(20) are utilized for calculating the second probability in (22) as

(24) $$\begin{array}{cc} STP_{2}^{DF} & =\mathrm{Pr}\left\{X\geq a_{1},\frac{\eta \delta \Lambda XY}{d_{X}^{m}d_{Y}^{m}}<R_{th}\right\}\\ \; & =\mathrm{Pr}\left\{X\geq a_{1},Y<\frac{a_{2}}{X}\right\} \end{array},$$

where $Y={\left|Y\right|}^{2}$, and $a_{2}=\frac{d_{X}^{m}d_{Y}^{m}R_{th}}{\eta \delta \Lambda }$.

Then, thanks to the PDF and CDF of the log-normally distributed RVs *X* and *Y*, the $STP_{2}^{DF}$ can be rewritten as

(25) $$\begin{array}{cc} STP_{2}^{DF} & =\int_{a_{1}}^{\infty }f_{X}\left(z\right)F_{Y}\left(\frac{a_{2}}{z}\right)dz\\ \; & =\int_{a_{1}}^{\infty }\frac{10\ln{\left(10\right)}^{-1}}{z\sqrt{8\pi \Omega_{X}^{2}}}\exp\left(-\frac{{\left(10\ln{\left(10\right)}^{-1}\ln\left(z\right)-2\omega_{X}\right)}^{2}}{8\Omega_{X}^{2}}\right)\\ \; & \;\times \left(1-Q\left(\frac{10\ln{\left(10\right)}^{-1}\ln\left(a_{2}z^{-1}\right)-2\omega_{Y}}{2\Omega_{Y}}\right)\right)dz. \end{array}$$

Eventually, (24) and (25) are substituted into (23) to obtain the STP of the HD-DF-PSR HWP system over log-normal fading channels as given in (21). The proof ends here. □

### 3.2. Opportunistic Amplify-and-Forward (OAF) Relaying Scheme

For the HD-AF-PSR HWP system, with the harvested energy in (13), R amplifies and forwards the signal from S to D. Thereby, the R’s transmitted signal can be obtained as follows:

(26) $$x_{r}\left(t\right)=\sqrt{\left(1-\delta \right)}\sqrt{\frac{1}{d_{X}^{m}}}GXs\left(t\right)+Gn_{r}\left(t\right),$$

where $E\left[{\left|s\right|}^{2}\right]=P_{S}$, and the relay gain of the HD-AF-PSR HWP system is obtained from

(27) $$\begin{array}{cc} G= & \sqrt{\frac{P_{R}}{\left(\frac{\left(1-\delta \right)P_{S}}{d_{X}^{m}}X+N_{0}\right)}} \end{array}$$

Accordingly, the signal that D receives is

(28) $$\begin{array}{cc} y_{d}\left(t\right)= & \sqrt{\frac{1}{d_{Y}^{m}}}Yx_{r}\left(t\right)+n_{d}\left(t\right)\\ = & \sqrt{\left(1-\delta \right)}\sqrt{\frac{1}{d_{X}^{m}d_{Y}^{m}}}GXYs\left(t\right)+\sqrt{\frac{1}{d_{Y}^{m}}}GYn_{r}\left(t\right)+n_{d}\left(t\right). \end{array}$$

Then, (16) and (27) are substituted into (28) and then manipulated to obtain the SNR at D as follows:

(29) $$\gamma_{di}^{AF}=\frac{\eta \delta (1-\delta )\Lambda XY}{\eta \delta d_{X}^{m}Y+\eta \delta \left(1-\delta \right)d_{X}^{m}Y+\left(1-\delta \right)d_{X}^{m}d_{Y}^{m}}.$$

In the HD-AF-PSR HWP system, the system’s instantaneous capacity is expressed as

(30) $$C_{d}=\frac{1}{2}\log_{2}\left(1+\gamma_{di}^{AF}\right).$$

**Theorem** **2.** *In the aforementioned context, the STP in the HD-AF-PSR HWP system can be formulated as*

(31) $$\begin{array}{cc} STP_{s,r,d}^{AF}\left(R_{th}\right)= & \frac{10\ln{\left(10\right)}^{-1}}{\sqrt{8\pi \Omega_{X}^{2}}}\int_{R_{th}\left(b_{2}+b_{3}\right)}^{\infty }\frac{1}{z}Q\left(\frac{10\ln{\left(10\right)}^{-1}\ln\left(\Gamma \right)-2\omega_{Y}}{2\Omega_{Y}}\right)\\ \; & \times \exp\left(-\frac{{\left(10\ln{\left(10\right)}^{-1}\ln\left(z\right)-2\omega_{X}-10\ln{\left(10\right)}^{-1}\ln\left(b_{1}\right)\right)}^{2}}{8\Omega_{X}^{2}}\right)dz \end{array},$$

*where $b_{1}=\eta \delta \left(1-\delta \right)\Lambda$, $b_{2}=\eta \delta d_{X}^{m}$, $b_{3}=\eta \delta \left(1-\delta \right)d_{X}^{m}$, $b_{4}=\left(1-\delta \right)d_{X}^{m}d_{Y}^{m}$, and $\Gamma =\frac{R_{th}b_{4}}{z-R_{th}\left(b_{2}+b_{3}\right)}$.*

**Proof.**  For more easily deriving the STP of the proposed HD-OAF-PSR HWP system, (29) is rewritten as

(32) $$\gamma_{d}^{AF}=\frac{b_{1}XY}{b_{2}Y+b_{3}Y+b_{4}},$$

with $b_{1}$, $b_{2}$, and $b_{3}$ given in (31).

Then, (32) is substituted to (8) to obtain

(33) $$\begin{array}{cc} STP_{s,r,d}^{AF} & =\mathrm{Pr}\left\{\frac{b_{1}XY}{b_{2}Y+b_{3}Y+b_{4}}\geq R_{th}\right\}\\ \; & =1-\mathrm{Pr}\left\{Y<\frac{R_{th}b_{4}}{b_{1}X-R_{th}\left(b_{2}+b_{3}\right)}\right\} \end{array}.$$

As *Y* is positive, the probability $P=\mathrm{Pr}\left\{Y<\frac{R_{th}b_{4}}{b_{1}X-R_{th}\left(b_{2}+b_{3}\right)}\right\}$ can be decomposed to

(34) $$P=\left\{\begin{array}{cc} \mathrm{Pr}\left\{Y\geq \frac{R_{th}b_{4}}{b_{1}X-R_{th}\left(b_{2}+b_{3}\right)}\right\}=1, & X>\frac{R_{th}\left(b_{2}+b_{3}\right)}{b_{1}}\\ \mathrm{Pr}\left\{Y<\frac{R_{th}b_{4}}{b_{1}X-R_{th}\left(b_{2}+b_{3}\right)}\right\}, & X<\frac{R_{th}\left(b_{2}+b_{3}\right)}{b_{1}} \end{array}\right.$$

The STP in (33) can be obtained from

(35) $$\begin{array}{cc} STP_{s,r,d}^{AF}= & 1-\left[\int_{0}^{z=\frac{R_{th}\left(b_{2}+b_{3}\right)}{b_{1}}}f_{X}\left(z\right)dz+\int_{z=\frac{R_{th}\left(b_{2}+b_{3}\right)}{b_{1}}}^{\infty }f_{X}\left(z\right)\mathrm{Pr}\left\{Y<\frac{R_{th}b_{4}}{b_{1}X-R_{th}\left(b_{2}+b_{3}\right)}\right\}dz\right] \end{array},$$

where $f_{X}\left(.\right)$ and $F_{Y}\left(.\right)$ represent, respectively, the PDF and the CDF of the log-normally distributed RVs *X* and *Y*. The two functions are given below:

(36) $$f_{X}\left(z\right)=\frac{10\ln{\left(10\right)}^{-1}}{z\sqrt{8\pi \Omega_{X}^{2}}}\exp\left(-\frac{{\left(10\ln{\left(10\right)}^{-1}\ln\left(z\right)-2\omega_{X}-10\ln{\left(10\right)}^{-1}\ln\left(b_{1}\right)\right)}^{2}}{8\Omega_{X}^{2}}\right),$$

and

(37) $$F_{Y}\left(\frac{R_{th}b_{4}}{z-R_{th}\left(b_{2}+b_{3}\right)}\right)=1-Q\left(\frac{10\ln{\left(10\right)}^{-1}\ln\left(\frac{R_{th}b_{4}}{z-R_{th}\left(b_{2}+b_{3}\right)}\right)-2\omega_{Y}}{2\Omega_{Y}}\right).$$

Consequently, (36) and (37) are substituted into (35) to obtain the STP of the DF-AF-PSR system as in (30). □

### 3.3. Opportunistic Decode-and-Forward and Amplify-and-Forward (ODF-AF) Relaying Scheme

Utilizing Theorem 1 and 2, the overall successful event is established with the help of the Selection Combining (SC) method, which combines the STP of the direct link and the ODF-AF relay-aided links to apply for the HWP system. It is noteworthy that the diversity order analysis is not affected with SC. Subsequently, from the condition of $\left\{\mathrm{good}\;\mathrm{direct}\;\mathrm{link}\right\}{⋃}_{k}\left\{\mathrm{good}\;k-\mathrm{th}\;\mathrm{ODF}-\mathrm{AF}\;\mathrm{relay}-\mathrm{aided}\;\mathrm{link}\right\}$, the below expression can be obtained:

(38) $$\begin{array}{cc} {\mathrm{STP}}^{sc}= & STP_{s,d}\times \prod_{k=1}^{K}\left\{\left(1-\chi_{k}\right)STP_{s,r,d}^{AF}+\chi_{k}STP_{s,r,d}^{DF}\right\}\\ = & A_{0}\times \prod_{k=1}^{K}\left(\left(1-\chi_{k}\right)\left(\int_{\gamma_{1}}^{\infty }A_{1}\left(x\right)\times A_{2}\left(x\right)\right)+\chi_{k}\left(A_{3}-\int_{\gamma_{2}}^{\infty }A_{4}\left(x\right)\right)\right) \end{array},$$

where

(39) $$A_{0}=Q\left(\frac{5}{\Omega_{Z}\ln\left(10\right)}\ln\left(\frac{R_{Z}}{\Lambda d_{Z}^{-m}}\right)-\frac{\omega_{Z}}{\Omega_{Z}}\right),$$

(40) $$A_{1}\left(x\right)=\frac{1}{x}Q\left(\frac{5}{\Omega_{Y}\ln\left(10\right)}\left(\frac{\left(1-\delta \right)d_{X}^{m}d_{Y}^{m}R_{th}}{x-\eta \delta d_{X}^{m}R_{th}\left(2-\delta \right)}\right)-\frac{\omega_{Y}}{\Omega_{Y}}\right),$$

-4.6cm0cm

(41) $$A_{2}\left(x\right)=\frac{5}{\ln\left(10\right)\sqrt{2\pi \Omega_{X}^{2}}}\exp\left(-\frac{{\left(10\ln{\left(10\right)}^{-1}\ln\left(x\right)-2\omega_{X}-10\ln{\left(10\right)}^{-1}\ln\left(\eta \delta (1-\delta )\Lambda \right)\right)}^{2}}{8\Omega_{X}^{2}}\right)$$

(42) $$A_{3}=Q\left(\frac{5}{\Omega_{X}\ln\left(10\right)}\ln\left(\frac{d_{X}^{m}R_{th}}{(1-\delta )\Lambda }\right)-\frac{\omega_{X}}{\Omega_{X}}\right),$$

(43) $$\begin{array}{cc} A_{4}\left(x\right)= & \frac{1}{x}\frac{5}{\ln\left(10\right)\sqrt{2\pi \Omega_{X}^{2}}}\exp\left(-\frac{{\left(10\ln{\left(10\right)}^{-1}\ln\left(x\right)-2\omega_{X}\right)}^{2}}{8\Omega_{X}^{2}}\right)\\ \; & \times \left(1-Q\left(\frac{5}{\Omega_{Y}\ln\left(10\right)}\ln\left(\frac{d_{X}^{m}d_{Y}^{m}R_{th}}{\eta \delta \Lambda }\frac{1}{x}\right)-\frac{\omega_{Y}}{\Omega_{Y}}\right)\right)dx, \end{array}$$

and $\varphi_{1}=\eta \delta d_{X}^{m}\left(2-\delta \right)R_{th}$, $\varphi_{2}=\frac{d_{X}^{m}R_{th}}{(1-\delta )\Lambda }$.

### 3.4. Throughput Performance

In this subsection, the throughput, τ, of delay-constrained HWP systems is determined. It is given that S transmits data with a constant rate of $R_{th}$ (bps/Hz) and the S–D effective communication time over the time block is T/2 over *T* (s). The HWP systems are subject to outage events owing to the log-normal fading effect on the wireless channels. Hence, the average throughput in the delay-constrained scenario is formulated in [29] as follows:

(44) $$\tau^{sc}=\frac{T/2}{T}\left(1-{\mathrm{OP}}^{sc}\right)R_{th},$$

where, thanks to Theorems 1 and 2, the outage probability, ${\mathrm{OP}}^{sc}$, in the multi-relay scenario is established utilizing (38) as ${\mathrm{OP}}^{sc}=\;\left(1-STP_{s,d}\right)\times {\left[\left(1-\chi_{k}\right)\left(1-STP_{s,r,d}^{AF}\right)+\chi_{k}\left(1-STP_{s,r,d}^{DF}\right)\right]}^{K}$.

## 4. Numerical Results and Discussion

This section presents the Monte Carlo simulation results from Matlab for the above analytical expressions. The parameters used for the simulation are listed in Table 1 below. With the exception of Figure 7, in all the figures, the number of relays is fixed at K=3. Moreover, the utilized STP henceforth stands for the STP of the HWP system where the direct and relay-aided links are combined.

**Figure 2:**
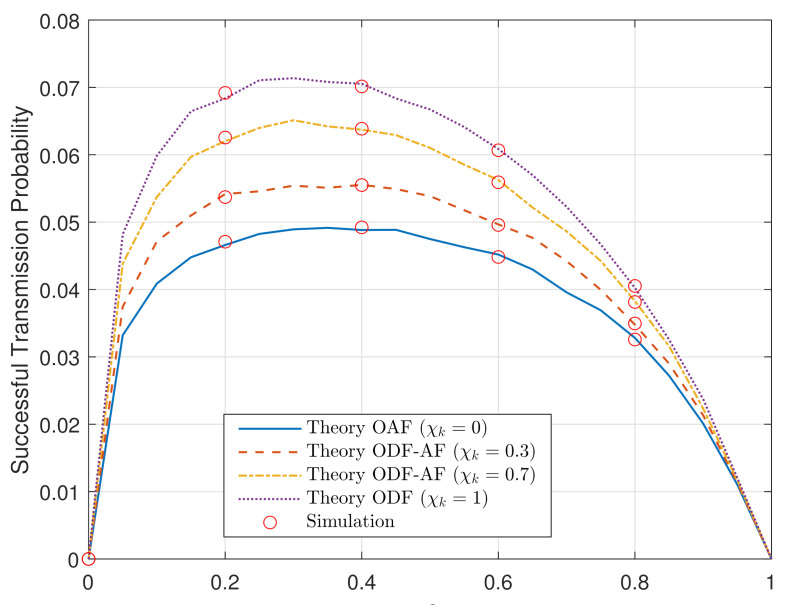
STP versus the PS ratio, δ, when $P_{S}$ = −10 (dB).

Figure 2 plots the overall analytical and simulated STP of the ODF-AF HWP systems concerning the EH PS ratio, δ, with four different decoding-state probabilities at the best-selected R, $\chi_{k}$. The power transmission at S is $P_{S}=-10$ (dB). The simulated results agree well with the analytical ones, proving the feasibility of the above derivations. As aforementioned, at $\chi_{k}=0$, the best R fully utilizes the ODF selection relaying protocol to decode and forward the message from S via direct link. At $\chi_{k}=1$, the OAF one is utilized and the best relay performs its task accordingly. Aside from the two extreme cases, in the two other ODF-AF protocol cases, $\chi_{k}=0.3$ and 0.7 are plotted as well. It can be observed that all the curves rise linearly and sharply as δ goes from 0 to approximately 0.05. Then, the STP values exponentially grow and peak with a lower pace as δ reaches 0.2 and then 0.4. Henceforth, all the STP curves converge exponentially to 0 as δ approaches 1.

**Figure 3:**
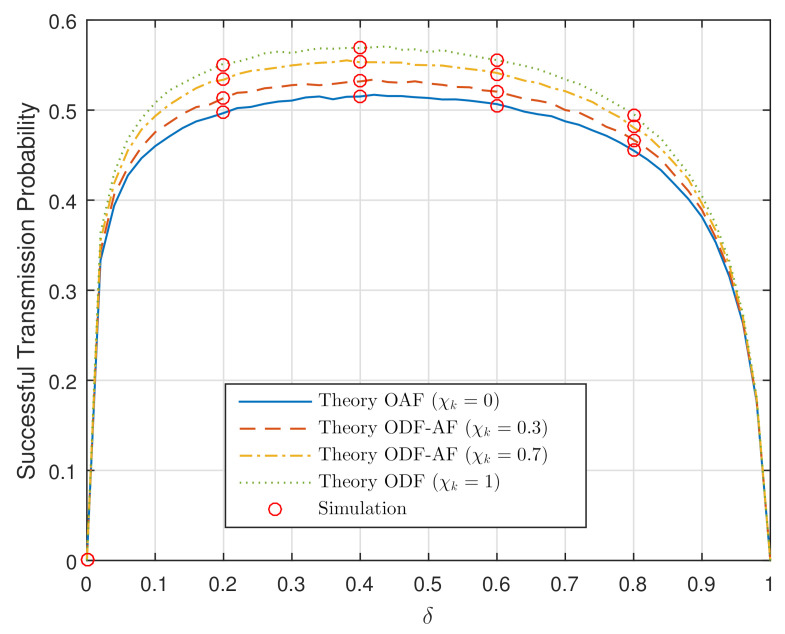
STP versus the PS ratio, δ, when $P_{S}$ = 1 (dB).

It is noteworthy in Figure 3 that as $P_{S}=1$ (dB), the STP has significantly improved and the curves become flatter. The STP remains peaked as **$\chi_{k}=0.4$** and has remarkably raised from 0.07 to approximately 0.67. This means that up to 40% of the power transmission $P_{S}=1$ (dB) can be allocated to the EH module to obtain the optimized performance. Indeed, the power transmission values used in Figure 2 and Figure 3 are rather small, −10 (dB) and 1 (dB). Given that the input power can be set to a higher value, the STP could accordingly raise to its maximum.

The fact that the STP curves drastically rise from and fall to 0 as δ, respectively, starts at 0 and approaches 1 is because the harvested energy in these two cases is either too small or undesirably too large that no resource is left for data transmission. In particular, the OAF delivers the lowest STP. Additionally, the probability to utilize the ODF becomes greater as $\chi_{k}$ value changes from 0.3 to 0.7 and finally 1. Subsequently, the higher the STP curve becomes, leading to better system performance. Optimizing the δ value is of importance for system performance and, thankfully, this task is realized in a thorough manner utilizing solely theory and Equation (15). Lastly, it is worth stating that the system performance can further be improved by unequally allocating the channels concerning the relative channel distributions.

**Figure 4:**
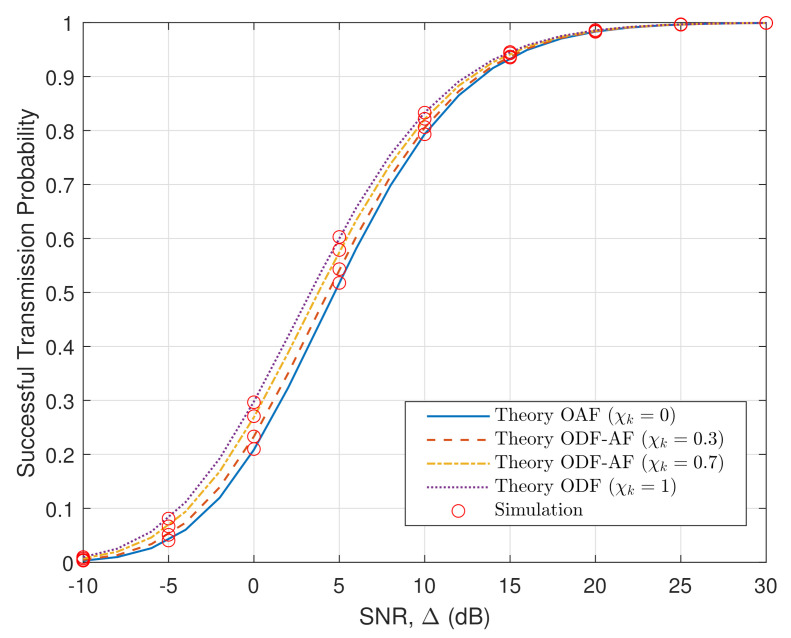
STP versus SNR.

Figure 4 presents the STP of the ODF-AF selection relaying scheme versus the SNR in the HWP system. The rise in the SNR logically results in better STP. The STP exponential curves rise rapidly as SNR approaches 10 (dB) and slowly converge to 1 as SNR grows further. As previously concluded, the ODF scheme performs better than the OAF one. This means that in the EH-PSR scenario, when SNR becomes larger, it is more beneficial to utilize the power to perform the ID task. Additionally, without spectrum sharing, the ODF-AF scheme performs better as the probability to utilize the ODF scheme increases.

**Figure 5:**
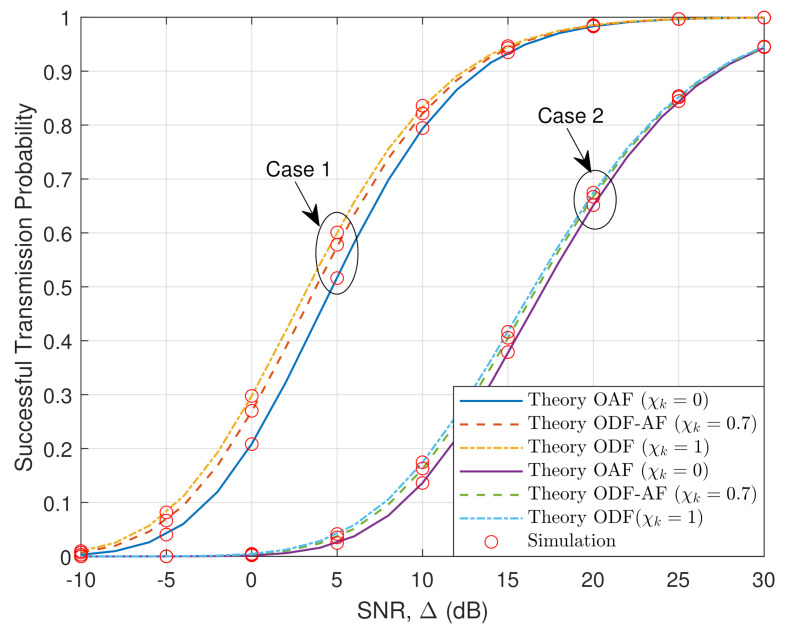
STP versus SNR in two different modes: Case 1 and Case 2.

**Figure 6:**
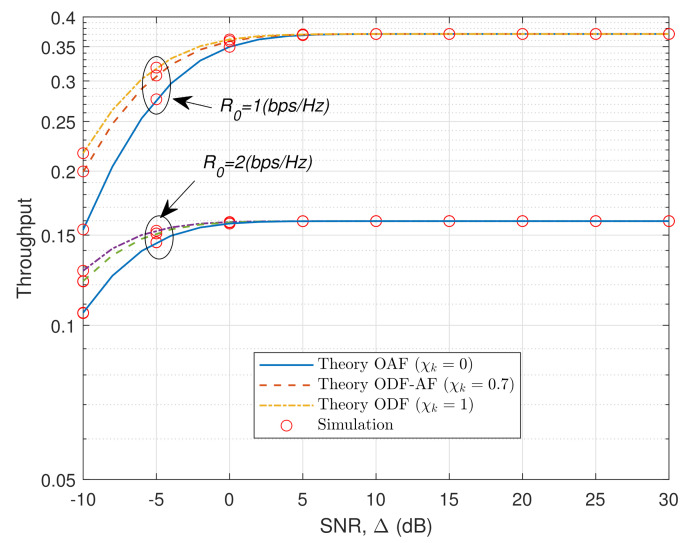
STP versus SNR with two different threshold values $R_{0}=1$ and $R_{0}=2$ (bps/Hz).

Figure 5 illustrates the STP of the ODF-AF-PSR scheme versus SNR in the HWP system, with two distance sets being Case 1: $d_{X}=d_{Y}=1$ (m); $d_{Z}=d_{X}+d_{Y}=2$ (m), and Case 2: $d_{X}=d_{Y}=2$ (m); $d_{Z}=d_{X}+d_{Y}=4$ (m). The trend of the curves in the two cases is the same as in Figure 4. It is obvious that Case 1 performs remarkably better than Case 2 owing to the shorter distances between terminals. This is because as the distances increase, the EH power and the signal strength that R receives are reduced significantly due to the path loss increase. Accordingly, the signal strength that D receives is also more attenuated, leading to the STP decrease. Figure 6 reveals the STP versus the SNR of the HWP system concerning two different cases of threshold values $R_{0}=1$ and 2 (bps/Hz). The OAF is represented with continuous lines, ODF-AF with dashed lines, and ODF with dash-dotted lines. As SNR increases from −10 (dB) to 0 (dB) for $R_{0}=2$, or 5 (dB) for $R_{0}=1$, the throughput rises exponentially, with ODF always being the highest. Nevertheless, after reaching their peaks, there is no difference in throughput as these curves remain at the same value regardless of the increase in the SNR. It is worth noting that the greater the opportunity to choose the ODF protocol, the higher the starting level of throughput, at SNR =−10 (dB).

**Figure 7:**
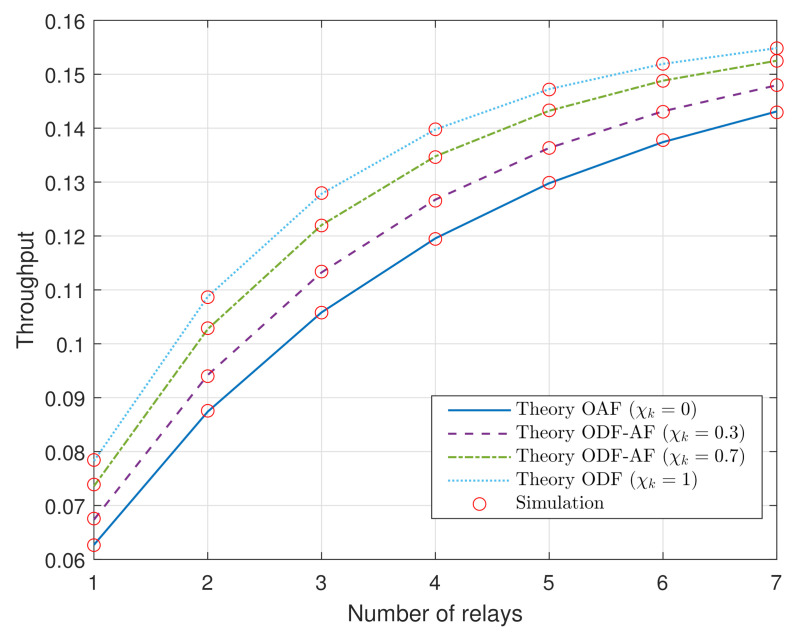
The throughput at D versus the number of R, *K*.

Figure 7 depicts the throughput analysis of the ODF-AF-PSR scheme at D versus the number of relays in the HWP system, *K*, with the power transmission of S being $P_{S}=-10$ (dB). Similar to what has been drawn previously, the ODF scheme has notably better performance than the OAF one. As there are more relays, the coding gain rises in association with the data transmission rates, leading to the throughput increase.

**Figure 8:**
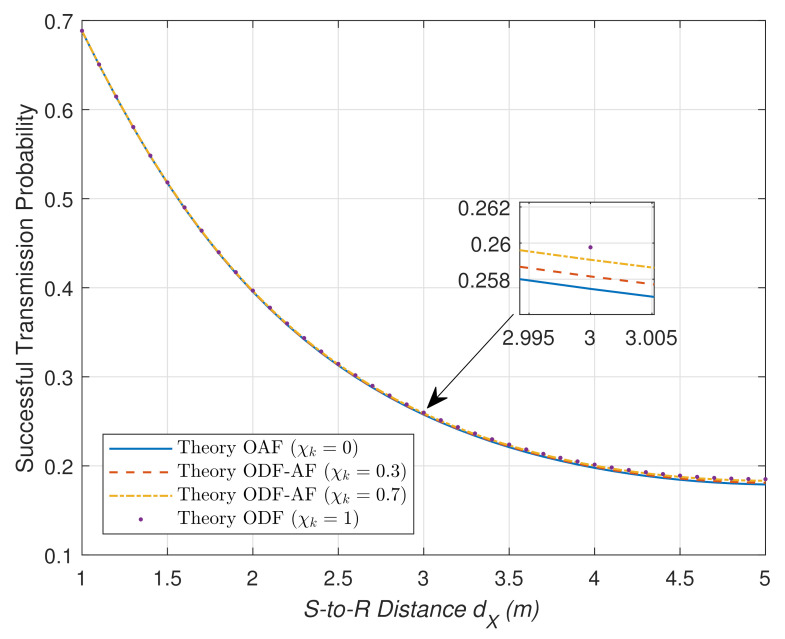
STP versus different S–R distance values, $d_{X}$, on condition that $d_{Z}=10$ (m), $d_{Y}=d_{Z}-d_{X}$.

Figure 8 plots the STP versus different values of $d_{X}$ of the HWP system given that $d_{Z}=10$ and $d_{Y}=d_{Z}-d_{X}$. It can be observed that the ODF theoretically performs better than OAF and ODF-AF no matter the location of R within the $d_{Z}$ range. Nonetheless, starting from the highest at (0.69), all the STP curves converge rapidly to (0.08) floor as R moves further to the midpoint of the S–D range. This is because as R moves towards the midway, it spends more time on EH; thus, the transmission time is shortened.

By utilizing the log-normal fading channels, the RF communication in an indoor scenario, through walls and with the presence of furniture and moving objects, was modeled in this study. The obtained simulation results show the system performance in terms of the STP of the proposed scheme in the HWP setting. Additionally, readers can make use of the simulation results to optimize the data rates without the need for additional bandwidth or power.

Lastly, what can be generally concluded from all the figures is that there is good agreement between the simulated and the analytical results. Thereby, the expressions derived herein can be utilized for future works.

## 5. Conclusions

To summarize, this manuscript aims at analyzing the behaviors of the opportunistic decode-and-forward and amplify-and-forward relay selection scheme in an energy harvesting power splitting-based relaying hybrid wireless and power line communication system over log-normal fading channels for indoor IoT networks. In particular, the issue of how to select an optimal relay for maximizing the signal-to-noise ratio that the destination receives is studied in terms of the successful transmission probability. Analytical expressions are derived and proven with relatively high accuracy thanks to Monte Carlo simulation results. The opportunistic decode-and-forward scheme is capable of delivering considerably better successful transmission probability in comparison with the opportunistic amplify-and-forward one. Additionally, the simulation results reveal that as the power splitting ratio and the source-to-relay distance increase, the system performance is subsequently degraded. The workflow presented in this manuscript can be followed by network designers who are interested in hybrid wireless and power line communication systems, given the assumptions and accuracy of the formulations. Lastly, the focus of future study will be the optimal hybrid decode-and-forward and amplify-and-forward protocol in cooperative relaying hybrid wireless and power line communication systems. Solving the newly emerging problems in combining and optimizing the two mature technologies as studied herein is promising as it brings about vast benefits, especially cost efficiency, for 5G smart home and smart city applications. 

## Figures and Tables

**Table 1:** Simulation parameters.

Primary Parameters	Description	Values
*W*	frequency bandwidth	5 (W)
$R_{0}$	transmission rate threshold	1 (bps/Hz)
$P_{S}$	traditional stabilized power source	−10 (dB)
$N_{0}$	overall AWGNs	1
η	energy harvesting efficiency	1
δ	power splitting fraction	0.2
*m*	path-loss	2.7
$d_{X}$	S to R distance	1 (m)
$d_{Y}$	R to D distance	1 (m)
$d_{Z}$	S to D distance	2 (m)
$\Omega_{X}$	S to R channel mean, log-normally distributed	4 (dB)
$\Omega_{Y}$	Rto D channel mean, log-normally distributed	4 (dB)
$\Omega_{Z}$	S to D channel mean, log-normally distributed	4 (dB)
$\omega_{X}$	S to R channel variance, log-normally distributed	3 (dB)
$\omega_{Y}$	R to D channel variance, log-normally distributed	3 (dB)
$\omega_{Z}$	S to D channel variance, log-normally distributed	3 (dB)
*K*	Number of relays	3

## Data Availability

Our study does not report any data.

## Abbreviations

Below are the abbreviations that are used in this manuscript:

5G	Fifth generation of cellular networks
EH	Energy harvesting
RF	Radio frequency
SWIPT	Simultaneous wireless information and power transfer
PLC	Power line communication
SG	Smart grids
IoT	Internet of Things
HWP	Hybrid wireless and power line communication
EE	Energy efficiency
SE	Spectrum efficiency
PT	Power transmission
PS	Power splitting
PSR	Power splitting-based relaying
TSR	Time splitting-based relaying
(O)DF	(Opportunistic) decode-and-forward
(O)AF	(Opportunistic) amplify-and-forward
ODF-AF	Opportunistic DF-AF
HD	Half-duplex
FD	Full-duplex
RS	Relay selection
SDP	Signal decision processor
SNR	Signal-to-noise ratio
CSI	Channel state information
AWGN	Additive white Gaussian noise
i.i.d.	independently and identically distributed
PDF	Probability density function
CDF	Cumulative distribution function
OP	Outage probability
STP	Successful transmission probability
ACK	Acknowledgement signal
RV	Random variable
TDMA	Time division multiple access
SC	Selection combining
S	The source node
D	The destination node
$R_{i}$	The i-th relay of *K* relays, (1≤i≤K)
*X*, *Y*, *Z*	The channel coefficients of S–$R_{i}$, $R_{i}$–D, S–D links
${\left|X\right|}^{2}$, ${\left|Y\right|}^{2}$ and ${\left|Z\right|}^{2}$	The i.i.d. log-normal RVs with $LN\left(2\omega_{X},4\Omega_{X}^{2}\right)$, $LN\left(2\omega_{Y},4\Omega_{Y}^{2}\right)$ and $LN\left(2\omega_{Z},4\Omega_{Z}^{2}\right)$
$\omega_{j}$, $\Omega_{j}^{2}$	The mean and the standard deviation of $10\log_{10}\left(j\right),j\in \left\{X;Y;Z\right\}$
$d_{X}$, $d_{Y}$, $d_{Z}$	The S–$R_{i}$, $R_{i}$–D, S–D distances
$P_{S}$	The power of S
$P_{R_{i}}$	The energy of *i*-th R
$n_{j}$	Additive white Gaussian noise (AWGN), ($j\in \left\{r_{k},d\right\}$)
$N_{0}$	The zero mean and variance at the *i*-th R and D
$\chi_{i}$	The decoding state of ODF-AF $R_{i}$, i∈1,⋯,K
